# Comparison of immune cells and diagnostic markers between spondyloarthritis and rheumatoid arthritis by bioinformatics analysis

**DOI:** 10.1186/s12967-022-03390-y

**Published:** 2022-05-04

**Authors:** Jiaqian Wang, Yuan Xue, Liang Zhou

**Affiliations:** 1 Department of Orthopaedic, Wuxi No.5 People’s Hospital, Wuxi, 214000 China; 2 Department of Orthopaedic, Wuxi Ninth People’s Hospital of Soochow University, Wuxi, 214000 China; 3 Department of Orthopaedic, Lianshui County Hospital, Huai’an, 223001 China

**Keywords:** Spondyloarthritis (SpA), Rheumatoid arthritis (RA), Immune cells, Diagnostic markers

## Abstract

**Background:**

Spondyloarthritis (SpA) and rheumatoid arthritis (RA) are chronic autoimmune diseases, but they are usually difficult to distinguish in the early stage of the diseases. The purpose of this study is to explore the differences of immune mechanism and diagnostic markers through bioinformatics analysis.

**Methods:**

First, microarray datasets from patients with SpA, RA and normal controls were obtained from the Gene Expression Omnibus (GEO) database. The differentially expressed genes (DEGs) between groups were identified in R software. Functional and pathway enrichment of DEGs were analyzed by David database. Then, we screened the hub genes using Cytoscape plugin, and constructed the protein–protein interaction (PPI) network and heatmap of hub genes. After that, CIBERSORT was used to evaluate the differences and connections of immune cells in SpA and RA, and screened out diagnostic markers. Correlation analysis was used to analyze the relationship between immune cells and diagnostic markers. Finally, quantitative real-time polymerase chain reaction (qRT‐PCR) was used to verify the effectiveness of immunodiagnostic markers.

**Results:**

We obtained three datasets, from which we can see that the functional enrichment of DEGs is mainly in cell chemotaxis, lymphocyte activation, primary immunodeficiency and other immune responses. The difference of immune cells between SpA, RA and normal control was concentrated in B, T lymphocytes cells, macrophages and dendritic cells. C19orf12 + S1PR3 is most associated with these immune cells and S1PR3 can be used as a diagnostic marker of this kind of immune diseases. In addition, MZB1 + XIST is closely related to T cells, NK cells and dendritic cells, and is expected to be used as a marker to distinguish the two diseases.

**Conclusion:**

Although the clinical manifestations of SpA and RA are similar, the pathogenesis is different. The screening of immune cells and diagnostic markers provides a more accurate target for the treatment of this kind of diseases.

**Supplementary Information:**

The online version contains supplementary material available at 10.1186/s12967-022-03390-y.

## Introduction

Chronic inflammatory rheumatism refers to the inflammation of joints, muscles and surrounding soft tissues with joint pain as the main manifestation. Among them, RA and SpA are the most common diseases that irreversibly damage joints, which seriously affect the life quality of patients [1]. However, due to the similar clinical manifestations and the lack of feasible biomarkers, this kind of disease is difficult to identify and diagnose. Current studies have found that immune factors play a vital role in the whole process, especially in the early stage [2, 3]. Therefore, finding new biomarkers and revealing immune mechanism are the key to early prevention and treatment.

The basic pathological changes of RA are chronic inflammation of synovium, pannus formation, and gradual destruction of articular cartilage and bone, resulting in joint deformity and loss of function [4]. At present, it is considered that HLA-DRB1 allele mutation is related to the disease [5]. In addition, abnormal immune regulation is also an important factor in the occurrence and development of RA. A large number of studies have shown that immune cells will infiltrate the joint synovium, such as activated CD4+ T cells, start a specific immune response and lead to the corresponding symptoms of arthritis [6]. However, CD8+ T cells have anti-inflammatory properties and may help to reduce the persistent autoimmune response of rheumatoid joints [7]. In addition, macrophages can secrete a large number of cytokines, chemokines and degrading enzymes, leading to joint inflammation and bone destruction [8]. Therefore, the study of immune cells in synovium is very important for the treatment of RA.

SpA, also known as serum negative spondyloarthritis, is a general term of chronic inflammatory rheumatism with the main manifestations of involving the spine and peripheral joints, or ligaments and tendons [9]. The disease has familial aggregation, but people with HLA-B27 gene do not necessarily suffer from the disease [10]. The etiology of SpA is not clear. Studies have shown that cytokines such as tumor necrosis factor α(TNF-α) and IL-17 can mediate the imbalance of immune and stromal cells, leading to bone remodeling [11]. However, the early diagnosis of SpA is difficult, and the research on immune cells is still limited [12]. So far, there is no study using CIBERSORT to analyze immune cells infiltration of patients with SpA.

In this study, we obtained DEGs between RA, SpA patients and normal controls. We not only analyzed its function enrichment, but also analyzed the relationship between immune cells. Most importantly, we also analyzed the differences of immune cells between the two diseases and screened diagnostic markers. This provides a direction for in-depth understanding of chronic inflammatory rheumatism and guiding diagnosis and treatment.

## Materials and methods

### Datesets download

We used “rheumatoid arthritis” or “spondyloarthritis” as keywords to search for element related datasets in GEO database (https://www.ncbi.nlm.nih.gov/geo/). The inclusion criteria are as follows: (1) *Homo sapiens* microarray analysis of RA and SpA with complete data; (2) Tissue samples were taken from the patient’s knee synovium; (3) Patients had no other immune diseases. Three eligible datasets were selected, GSE41038 was used to compare SpA and normal controls. GSE12021 included twelve RA patients and four normal controls for comparison. The comparison between RA and SpA used dataset GSE30023.

### Identification of DEGs

The difference analysis was carried out by microarray data linear model (Limma) software package. The *p* value less than 0.05 and |log2-fold change (FC)| > 1 were considered to be statistically significant. The results of DEGs are presented by volcano map. The PPI network of DEGs was predicted using online tool STRING (https://string-db.org/). Network diagram uses Cytoscape software (v3.8.0) to achieve better visualization.

### Screening of hub genes

Hub gene has more connections in PPI network and usually plays an important role in diseases. Cytohubba is a built-in tool in Cytoscape, which uses different methods to identify hub genes in the network. We calculate the top 20 hub genes by degree, maximum cluster centrality (MCC) and maximum neighborhood component (MNC). The software package “Heatmap” is used to visualize the up and down regulation of hub genes.

### Functional enrichment analysis

We used two different methods for enrichment analysis to improve accuracy. Gene set enrichment analysis (GSEA) compared the differential expression of all genes in the two types of samples. However, the analysis of Gene Ontology (GO) and Kyoto Encyclopedia of Genes and Genomes (KEGG) pathway enrichment was only aimed at the differential genes. *p* < 0.05 is considered to be statistically significant. The enrichment pathways and functions were visualized by ggplot2 package.

### Evaluation of immune cells

CIBERSORT can transform the standardized gene expression matrix into the composition of invasive immune cells. Upload data to CIBERSORT (https://cibersort.stanford.edu/). The website defined 22 components of infiltrating immune cells using LM22 characteristic matrix, and only the data with *p* value < 0.05 were retained. Violin diagrams are used for visualization. Psych package is used to calculate the correlation coefficients of various immune cells.

### Principal component analysis (PCA)

We used GraphPad Prime 9 for PCA cluster analysis of gene matrix data. The intra group data repeatability of the dataset was tested by PCA cluster analysis. Using the same analysis of immune cell infiltration matrix data, two-dimensional PCA clustering results were obtained.

### Predictive biomarkers and value analysis

Overlapping genes between datasets serve as potential diagnostic markers, as shown in the Venn diagram. Receiver operating characteristic (ROC) curves were performed by GraphPad Prime 9 to predict the diagnostic effectiveness of biomarkers. The area under ROC curve (AUC) was calculated. AUC > 0.8 showed that biomarkers had good diagnostic value.

### Correlation analysis

The correlation of the identified diagnostic biomarkers with the levels of infiltrating immune cells was explored using Spearman’s rank analysis. Use the bubble chart and “ggplot2” package to visualize the results.

### qRT‐PCR and statistical analysis

Based on the results of the above analysis, the synovium samples of 3 patients with RA and 3 patients with SpA were obtained from knee arthroscopy, and the synovium of 3 patients with meniscus injury was used as control to verify the expression levels of six diagnostic markers. Total RNA was extracted from synovial membrane using the TRIzol reagent (Beyotime, China) according to the manufacturer’s instruction, and reversely transcribed. QRT-PCR was performed on CFX connect real-time PCR detection system (Bio-rad, USA). The relative gene expressions were calculated by the 2^−ΔΔCt^ method. GAPDH was selected to normalize the expression levels of the target genes. All experiments were performed independently in triplicate.

The sequences of specific primers are as follows: C19orf12 (5′-ATCGGTTACGGATCGAACA-3′), ALPK2 (5′‐GCGAAGACCTTGGCATTTATT‐3′), S1PR3 (5′-GTGATCCTCTACGCACGCATC-3′), MZB1 (5′-CTCACAGGCCCAGGACTTAG-3′), XIST (5′-CTCTCCATTGGGTTCAC-3′), CCDC88C (5′‐TCTGGTGACCTGGGTGAAAA‐3′) and GAPDH (5′‐CCGTTGAATTTGCCGTGA‐3′).

## Results

### DEGs identification

**Fig. 1:**
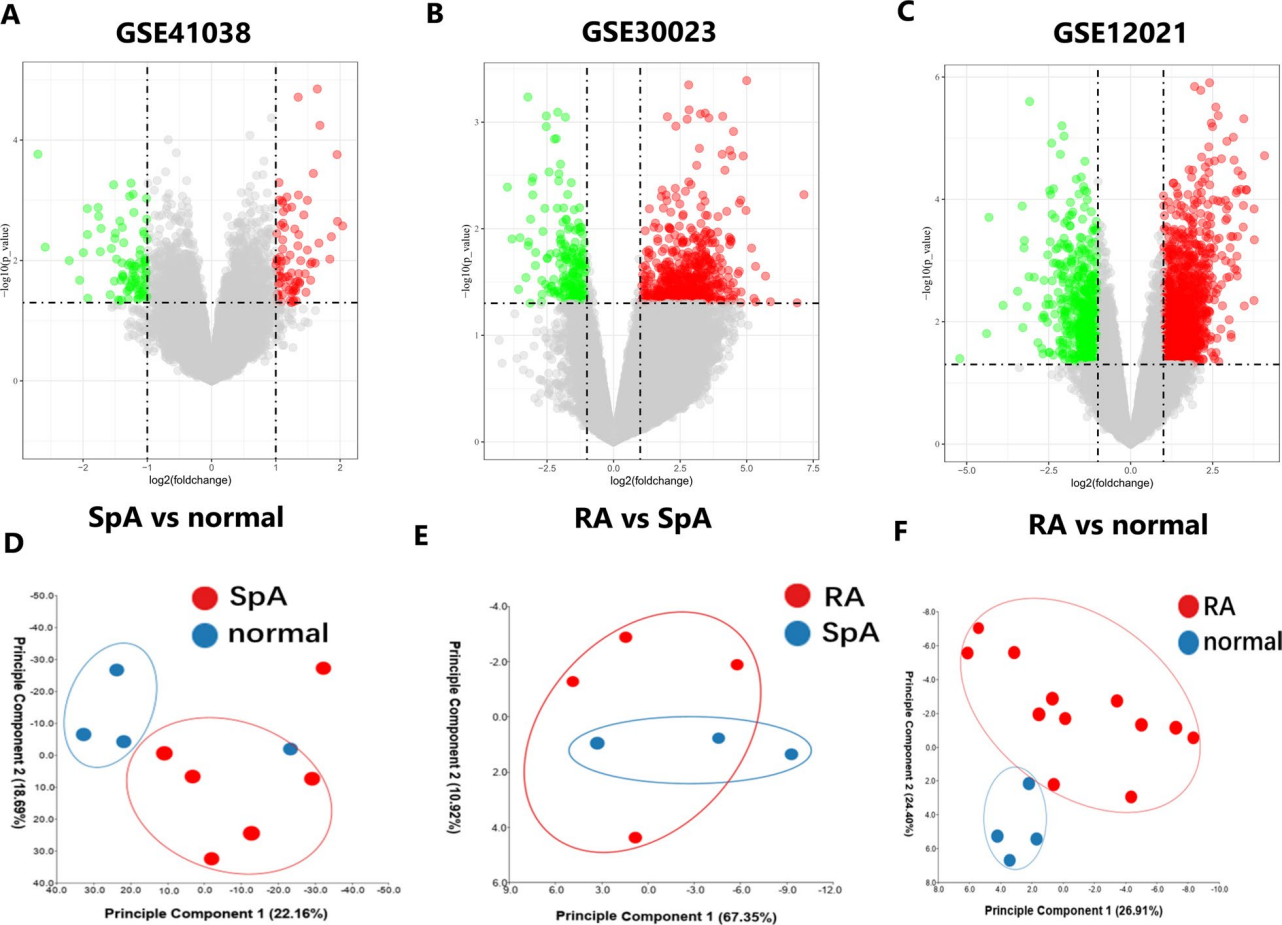
Differentially expressed genes identification. **A** Volcano map of GSE41038. **B** Volcano map of GSE30023. **C** Volcano map of GSE12021. **D** PCA cluster plot of GSE41038. **E** PCA cluster plot of GSE30023. **F** PCA cluster plot of GSE12021

GSE41038 contained synovial tissue of 6 SpA and 4 normal controls, and GSE12021 contained synovial tissue of 12 RA and 4 normal controls. GSE30023 was used for validation and contained synovial tissue from 4 RA and 3 SpA patients. After the microarray was standardized with *p* values < 0.05 and |log2 fold change (FC)| > 1, 151 genes were found in GSE41038 dataset, 425 genes were found in GSE30023 dataset and 1219 genes were found in GSE12021 (Fig. 1A–C). PCA cluster analysis of the dataset showed that there were significant differences between RA, SpA tissues and normal control tissues, while there were similarities between RA and SpA tissues, which can be used for follow-up research (Fig. 1D–F).

### Hub genes screening

**Fig. 2:**
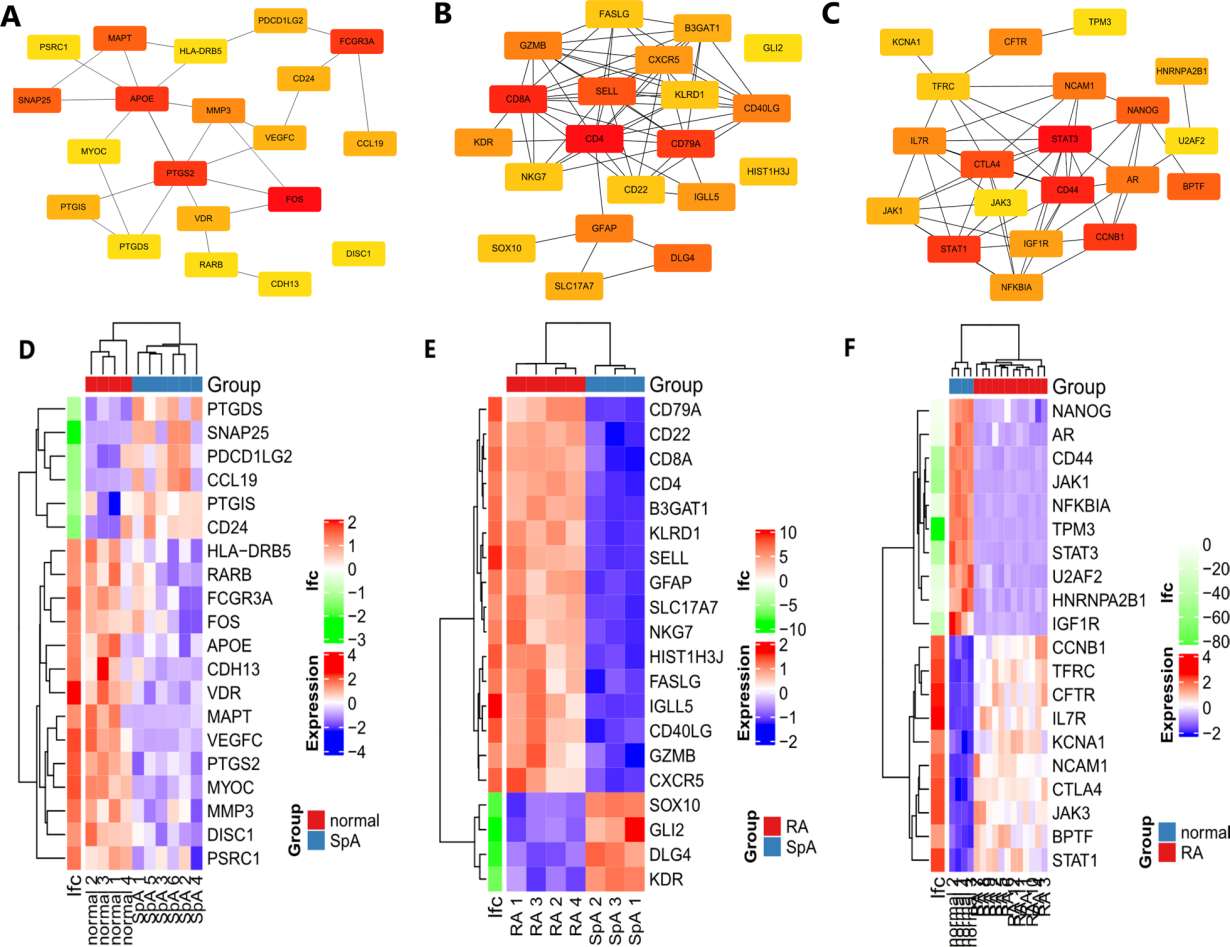
Hub genes screening. **A** PPI network of GSE41038 hub genes. **B** PPI network of GSE30023 hub genes. **C** PPI network of GSE12021 hub genes. **D** Heatmap of GSE41038 hub genes. **E** Heatmap of GSE30023 hub genes. **F** Heatmap of GSE12021 hub genes

Firstly, we constructed the interaction network between DEGs coding proteins through Cytoscape. The GSE41038 network consists of 97 nodes and 123 edges. Among them, 77 up-regulated genes were marked in red and 74 down-regulated genes were marked in blue (Additional file 1: Fig. S1). The GSE12021 PPI network consists of 871 nodes and 2292 edges. 769 up-regulated genes are marked in red and 450 down-regulated genes are marked in blue (Additional file 2: Fig. S2). The DEGs network of RA and SpA contains 211 nodes and 442 edges. 310 up-regulated genes and 115 down-regulated genes (Additional file 3: Fig. S3). Then, according to degree, MCC and MNC, we use Cytohubba to identify hub genes. For the three datasets, we have identified 20 hub genes respectively, which are displayed by the PPI network diagram (Fig. 2A–C). Finally, the expression of hub genes is shown by the heatmap (Fig. 2D–F).

### Enrichment analysis between SpA and normal

**Fig. 3:**
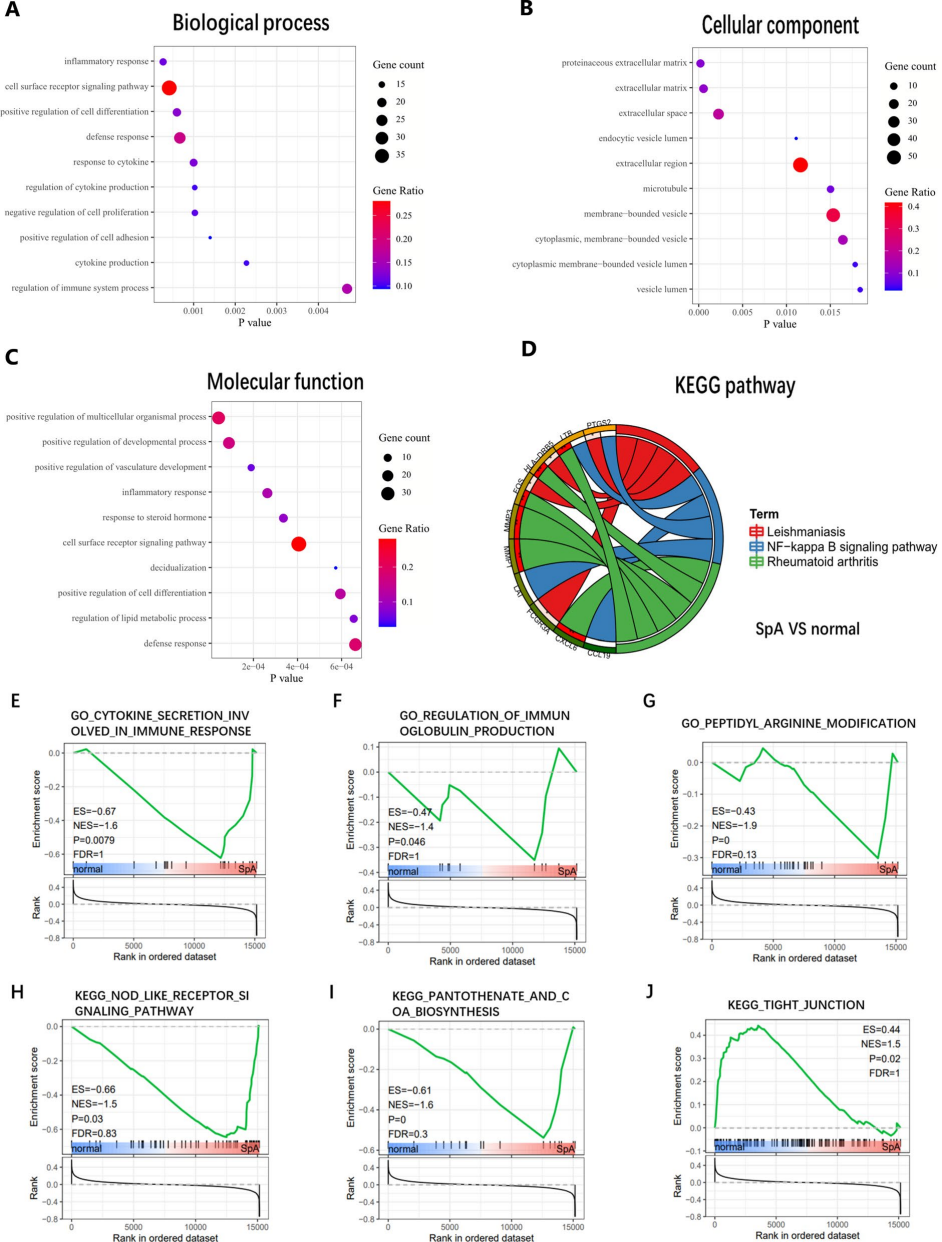
Enrichment analysis between SpA and normal. **A** Biological process of DEGs. **B** Molecular function of DEGs. **C** Cellular component of DEGs. **D** KEGG pathway of DEGs. **E**–**G** GSEA of biological processes. **H**–**J** GSEA of KEGG pathway

GO enrichment analysis showed that the changes of biological process (BP) of DEGs were mainly concentrated in inflammatory response, cell differentiation and the regulation of chemokines. KEGG pathway analysis showed that leishmaniasis, NF-kB signaling pathway and rheumatoid arthritis were statistically significant (Fig. 3A–D). GO and KEGG enrichment analysis focuses on comparing the gene expression differences between the two groups. GSEA analyzes the whole gene dataset and will not omit genes with insignificant differential expression but of great significance. Statistically significant biological processes are mainly concentrated in arginine and threonine modification, chemokine secretion, immunoglobulin production and so on (Fig. 3E–G). In addition, the top enriched pathways include NOD like receptor signaling pathways, pantothenate and CoA biosynthesis and tight junction (Fig. 3H–J).

### Enrichment analysis between RA and normal

**Fig. 4:**
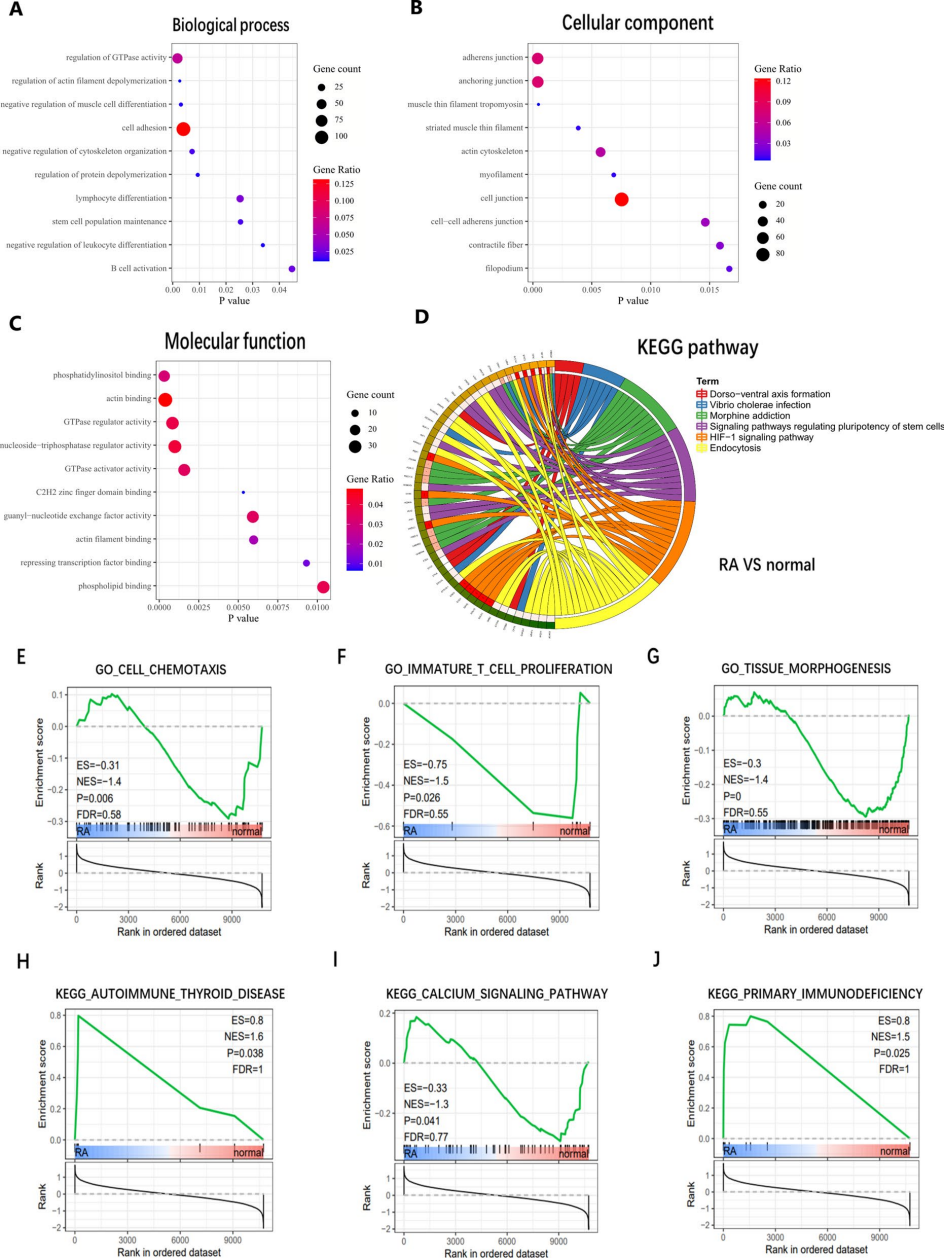
Enrichment analysis between RA and normal. **A** Biological process of DEGs. **B** Molecular function of DEGs. **C** Cellular component of DEGs. **D** KEGG pathway of DEGs. **E**–**G** GSEA of biological processes. **H**–**J** GSEA of KEGG pathway

The same analysis was carried out between RA and normal control group. The biological process and molecular function enrichment were closely related to GTPase activity. Significant enrichment of GO biological processes in various cell differentiation, such as leukocytes, lymphocytes, B cells, etc. In addition, HIF-1, regulating pluripotency of stem cells and endocytosis are considered to be the most remarkably enrichment pathways (Fig. 4A–D). GSEA results showed that immune factors such as cell chemotaxis, T cell proliferation and primary immune deficiency were related to RA (Fig. 4E–J).

### Enrichment analysis between RA and SpA

**Fig. 5:**
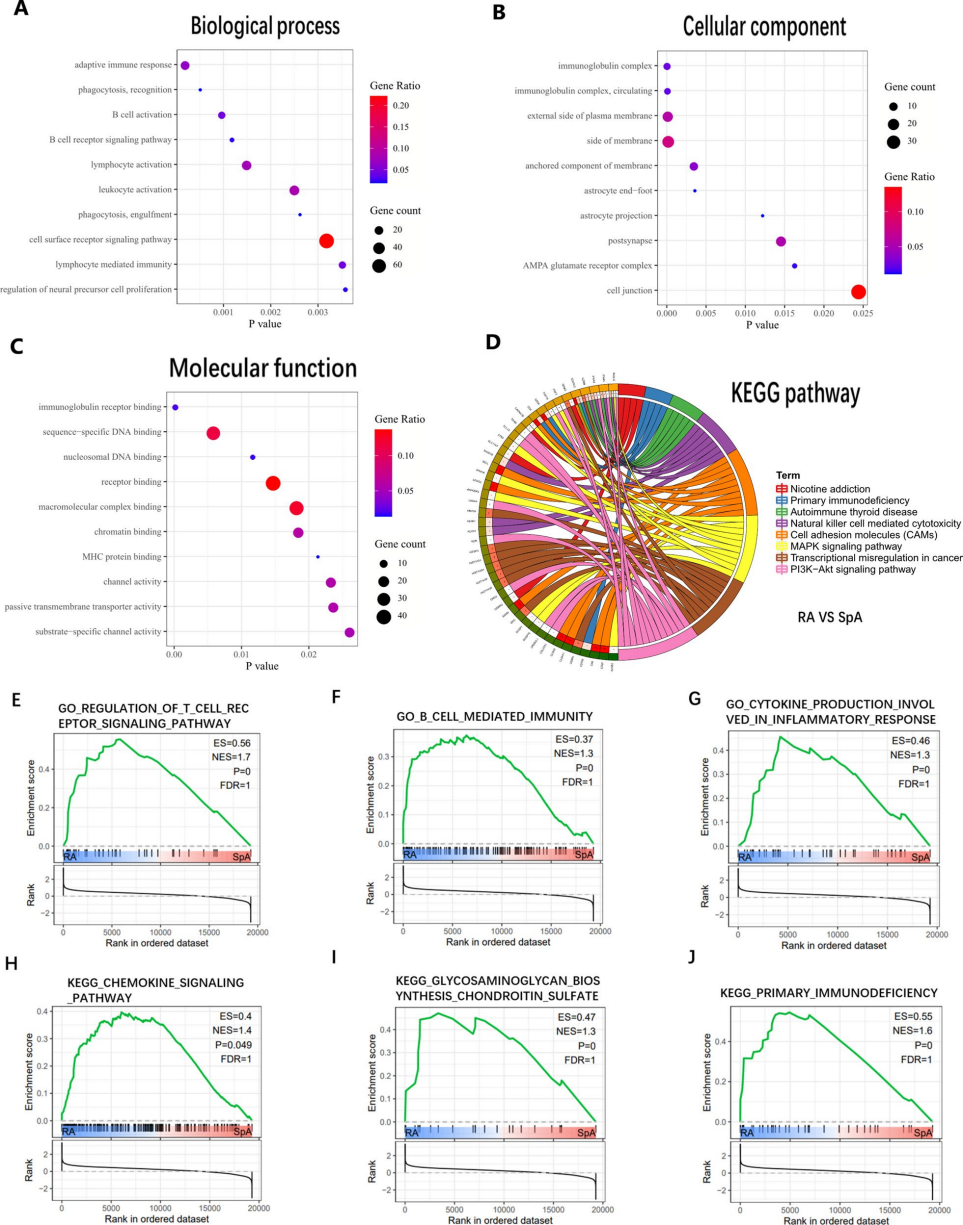
Enrichment analysis between RA and SpA. **A** Biological process of DEGs. **B** Molecular function of DEGs. **C** Cellular component of DEGs. **D** KEGG pathway of DEGs. **E**–**G** GSEA of biological processes. **H**–**J** GSEA of KEGG pathway

We are most concerned about the differences between RA and SpA. DEGs are mainly related to biological processes such as B cells activation and cell phagocytosis. Immunoglobulin is the most significant enrichment of cellular components and molecular functions. In addition, DEGs are associated with MAPK, PI3K-Akt, primary immune deficiency, and natural killer cell-mediated cytotoxic signaling pathways (Fig. 5A–D). GSEA analysis verified this result and found that T cells and chemokines were different between the two diseases (Fig. 5E–J).

### Infiltration of immune cells results

**Fig. 6:**
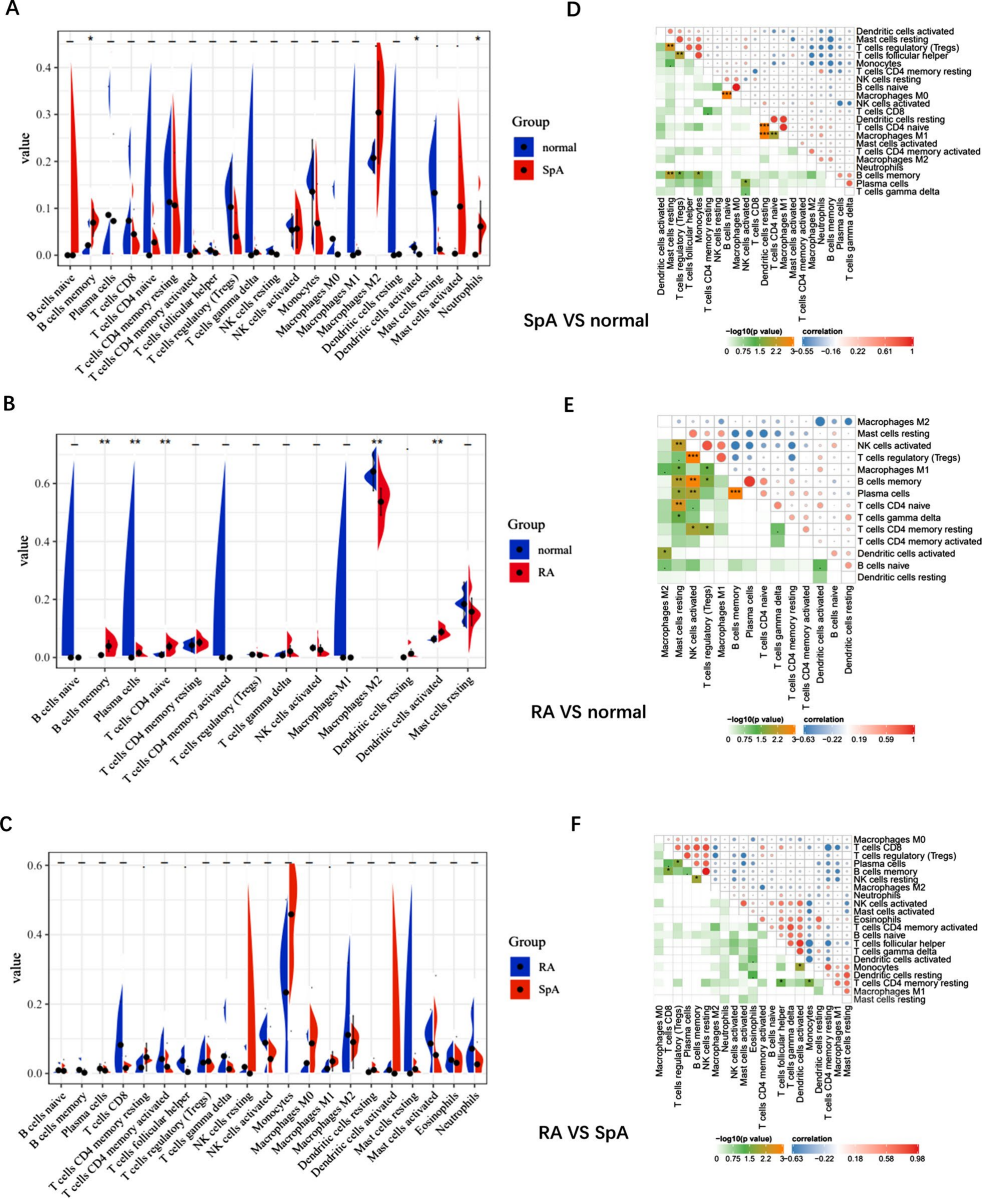
Evaluation of immune cells. **A** Violin diagram of GSE41038 immune cells. **B** Violin diagram of GSE12021 immune cells. **C** Violin diagram of GSE30023 immune cells. **D** Correlation heatmap of GSE41038 immune cells. **E** Correlation heatmap of GSE12021 immune cells. **F** Correlation heatmap of GSE30023 immune cells

In order to further explore the differences of immune cells between the two diseases, CIBERSORT algorithm was used for verification. Under the standard of *p* < 0.05, SpA samples contain a high proportion of B cells memory and neutrophils, while the proportion of activated dendritic cells is relatively low. Compared with normal samples, RA samples contained a higher proportion of B cells memory, plasma cells, T cells CD4 naive and activated dendritic cells, while the proportion of M2 macrophages was relatively low (*p* < 0.05). Violin diagram showed that compared with RA, the SpA group had more T cells CD4 memory resting, M1 macrophage infiltration and less T cells follicular helper (Fig. 6A–C). In addition, according to the correlation coefficient of immune cells, B cells naïve were positively correlated with M0 macrophages, dendritic cells resting were positively correlated with M1 macrophages and T cells CD4 naive (SpA vs. normal). Plasma cells and B cells memory, NK cells activated and T cells regulatory were positively correlated (RA vs. normal). The correlation between immune cells in SpA and RA groups was relatively low (Fig. 6D–F).

### Correlation between biomarkers and immune cells

**Fig. 7:**
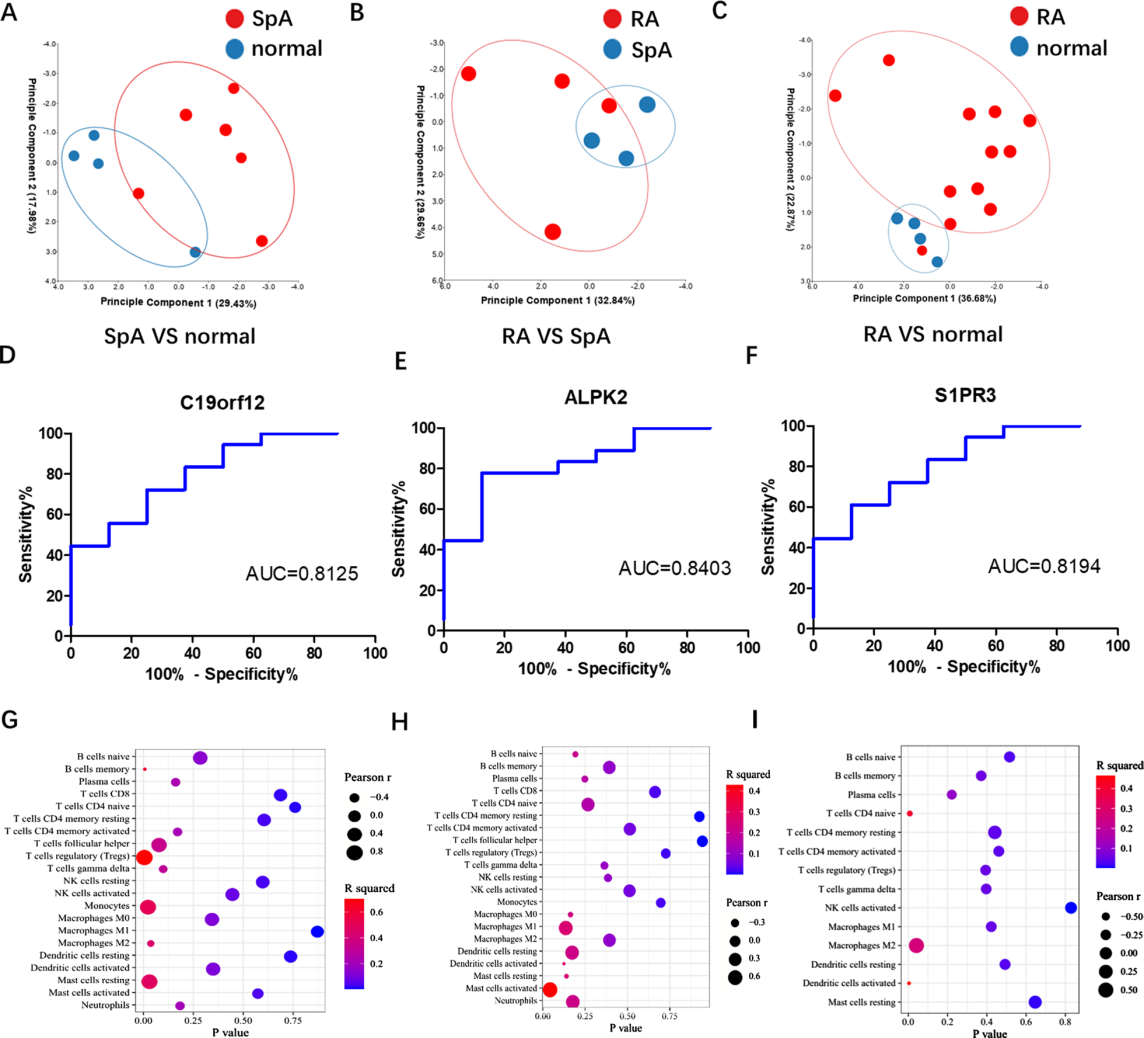
Correlation between diagnostic markers and immune cells. **A** PCA cluster plot of GSE41038 immune cells. **B** PCA cluster plot of GSE30023 immune cells. **C** PCA cluster plot of GSE12021 immune cells. **D** ROC curve of C19orf12. **E** ROC curve of ALPK2. **F** ROC curve of S1PR3. **G** C19orf12 and immune cells. **H** ALPK2 and immune cells. **I** S1PR3 and immune cells

The PCA clustering results of immune cells showed that there were differences among the three groups (Fig. 7A–C). The overlap among the GSE12021 and GSE41038 contained 4 genes, one of which is the DEGs of GSE30023, which should be removed (Additional file 4: Fig. S4). The AUCs of the three diagnostic markers were C19orf12 (AUC = 0.8125), ALPK2 (AUC = 0.8403) and S1PR3 (AUC = 0.8194), which had diagnostic value (Fig. 7D–F). According to the correlation analysis, the markers related to immune cells were screened, and the results showed that C19orf12 was positively correlated with T cells regulatory (r = 0.8371, *p* = 0.0025) and mast cells resting (r = 0.6877, *p* = 0.028), negatively correlated with B cells memory (r = − 0.7905, *p* = 0.0065) and M2 macrophages (r = − 0.6653, *p* = 0.0358). S1PR3 was positively correlated with T cells CD4 naive (r = 0.6389, *p* = 0.0077), M2 macrophages (r = 0.5145, *p* = 0.0414) and negatively correlated with activated dendritic cells (r = − 0.6797, *p* = 0.0038). Under the criteria of r > 0.4 and *p* < 0.05, ALPK2 was only related to activated mast cells (Fig. 7G, I).

**Fig. 8:**
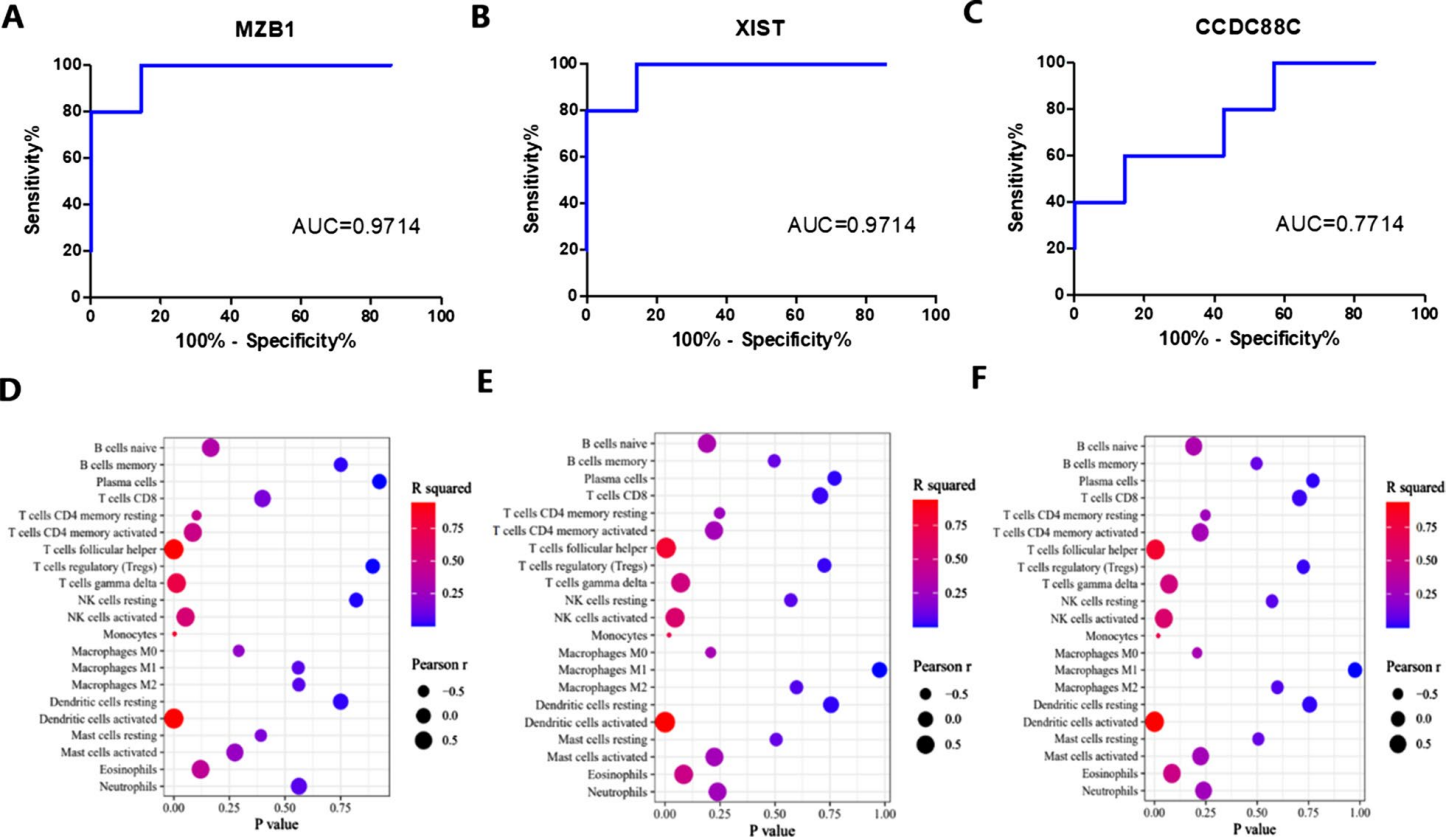
Diagnostic markers of RA vs. SpA. **A** ROC curve of MZB1. **B** ROC curve of XIST. **C** ROC curve of CCDC88C. **D** MZB1 and immune cells. **E** XIST and immune cells. **F** CCDC88C and immune cells

There are 36 DEGs in GSE30023, of which 4 genes overlap with GSE12021. However, STXBP6 has nothing to do with immune cells. MZB1, XIST, CCDC88C are highly correlated with T cells follicular helper, T cells gamma delta, NK cell activated, dendritic cells and monocytes (Fig. 8D–F). Moreover, MZB1 (AUC = 0.9714) and XIST (AUC = 0.9714) values > 0.08 can be used as diagnostic markers to distinguish the two diseases (Fig. 8A–C).

### qRT‐PCR validation of diagnostic markers

**Fig. 9:**
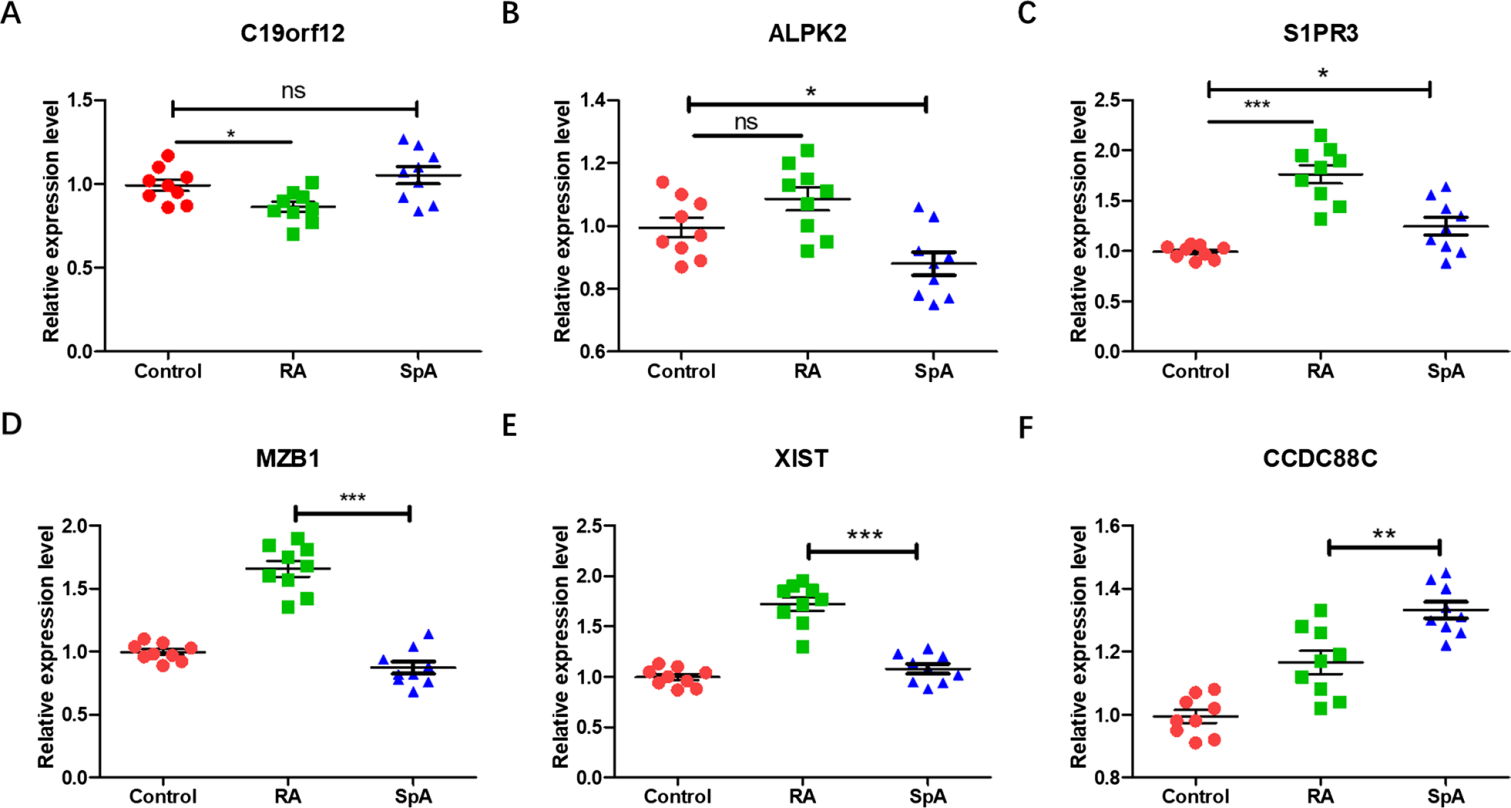
MRNA expression level of diagnostic markers. **A** Expression level of C19orf12. **B** Expression level of ALPK2. **C** Expression level of S1PR3. **D** Expression level of MZB1. **E** Expression level of XIST. **F** Expression level of CCDC88C (*p < 0.05, **p < 0.01, ***p < 0.001)

The results of qRT‐PCR showed that S1PR3 was significantly up-regulated in synovium of RA and SpA patients (*p* < 0.05). Compared with the control group, there was no significant difference in the expression levels of C19orf12 and ALPK2 (Fig. 9A–C). In addition, there were significant differences in the expression of MZB1, XIST and CCDC88C between RA and SpA groups. Among them, MZB1 and XIST were significantly up-regulated in the synovium of RA patients (*p* < 0.001), and CCDC88C was significantly down-regulated (Fig. 9D–F). These results confirmed the effectiveness of immunodiagnostic markers.

## Discussion

RA and SpA are the most common chronic inflammatory diseases causing multi joint pain. Because of the continuous destruction of joints, early diagnosis and treatment are particularly important [13]. However, the clinical manifestations of early diseases are similar, the diagnosis time is too long, and there is no effective biomarker, especially the negative serum rheumatoid factor and anti-cyclic citrulline antibody, which bring great difficulties to the treatment [14]. Increasing number of studies have shown that the inflammatory microenvironment and inflammatory cells of synovium play an indispensable role in diseases [15, 16]. Therefore, it is of great significance to study the infiltrating immune cells in synovial tissue and find new differential diagnostic markers.

Compared with the normal control group, the number of B cells memory, plasma cells, T cells CD4 naive and activated dendritic cells in RA synovium increased significantly, and the number of M2 macrophages decreased significantly. Previous studies have shown that B cells activate and differentiate into plasma cells, secrete a large amount of immunoglobulin and form a complex with rheumatoid factor, which can induce inflammation after complement activation [17]. Special components in synovial tissue and endogenous substances produced in vivo can also be presented by dendritic cells as self-antigens, activate CD4+ T cells and lead to inflammation [18]. T cells follicular helper (Tfh) is a subtype of CD4+ T cells, which can help B cells and regulate the production of antibodies, so as to further participate in the occurrence of RA [19]. In addition, inducing anti-inflammatory M2 macrophages, inhibiting the production of inflammatory factors and alleviating synovitis of RA are also the focus of current research. This is consistent with our experimental results [20]. Enrichment analysis also showed that cellular immune processes such as B cells differentiation, T cells proliferation, cell adhesion, cell chemotaxis and endocytosis were involved in the pathogenesis of RA. The above results show that B cells, T cells, dendritic cells and macrophages are the key cells in the occurrence and development of RA.

Many patients with SpA first show swelling and pain of peripheral joints, and then appear symptoms of low back pain several years later. The lack of specific laboratory test indicators has brought great difficulties to disease diagnosis [21]. It can be seen from our experiment that SpA has more B cells memory, neutrophils and less activated dendritic cells than normal control. There are also differences between M2 macrophages and mast cells. Current studies have shown that dendritic cells overproduce cytokines and migrate to potential inflammatory sites, where both immune cells of the innate immune system and cells of the adaptive immune system are activated to produce more pro-inflammatory cytokines. These cytokines can in turn interact with receptors on effector cells, such as macrophages and neutrophils, leading to tissue destruction [22, 23]. At the same time, we also found that SpA is highly similar to RA in the direction of functional enrichment such as cell chemotaxis and immunoglobulin production. More interestingly, the DEGs enrichment pathway of SpA is related to RA. The two diseases are difficult to distinguish, especially in the early stage of the disease. Although the results of immune cells infiltration showed that there was no significant difference between the two diseases, DEGs were enriched in B cells related functions. GSEA was also significantly enriched in B cells and T cells related immune regulation. Previous SpA studies have found that innate immune system activation seems to be more important than more typical adaptive immune system diseases such as RA [24]. Our pathway analysis also showed that innate immunity, such as natural killer cell-mediated cytotoxicity and primary immunodeficiency, were significantly enriched. These results suggest that SpA may be related to innate immune cells such as NK cells, and the number of T cells, B cells is less than RA.

In order to further study the diagnostic markers of chronic rheumatoid arthritis, we finally screened the same three genes (C19orf12, ALPK2, S1PR3) in the DEGs between the two diseases and normal controls. ROC regression analysis found that the three genes had good specificity and sensitivity, but only two had high correlation with immune cells (C19orf12, S1PR3). Among them, C19orf12 plays an important role in the immune cell infiltration of SpA, and S1PR3 is closely related to the immune cell infiltration of RA. S1PR3 is a bioactive sphingolipid that regulates signaling pathways essential to biological processes, including cell growth, immune cell transport and inflammation [25]. In our study, we found that the expression of S1PR3 in RA patients was increased, which was consistent with the experimental results of Takuya. Inhibiting S1PR3 can reduce the production of pro-inflammatory cytokines and bone destruction, so as to treat autoimmune arthritis [26]. So far, the exact cellular function of C19orf12 and its relationship with immune diseases are not clear. This protein is commonly expressed, but especially in the brain, blood cells and adipocytes [27]. Through the verification of qRT-PCR, we finally determined S1PR3 as a biomarker for the early diagnosis of immune arthritis.

In addition, four genes were screened from DEGs of SpA and RA, three of which were highly related to immune cells (MZB1, XIST, CCDC88C). These three genes have been confirmed to be related to the occurrence of RA [28–30]. MZB1 plays an important role in humoral immune response and is related to a variety of immune cells. It can enhance the ability of B cells to differentiate into plasma cells, which is the same as the pathogenesis of RA [31]. XIST leads to RA by inhibiting cell proliferation and inducing apoptosis, and is considered as a diagnostic marker [32]. Neither of these two genes has been reported to be associated with SpA and has high diagnostic value, so we use them as markers to distinguish RA from SpA. In addition, these three genes are related to T cells follicular helper, T cells gamma delta, NK cell activated, dendritic cells and monocytes. These immune cells may be considered as differential cells of the two diseases.

There are still many limitations in this experiment. In the future, we will collect more synovial samples from RA and SpA patients, detect the differences of various immune cells by flow cytometry, and obtain more accurate diagnostic markers.

## Conclusion

In conclusion, the occurrence of chronic inflammatory rheumatism is related to B, T lymphocytes cells, macrophages and dendritic cells. S1PR3 is most related to these immune cells and can be used as a diagnostic marker of such immune diseases. In addition, the different expressions in RA and SpA may be T cells, NK cells and dendritic cells. MZB1 + XIST is expected to be used as a diagnostic marker to distinguish the two diseases. This study provides a more accurate index and updated perspective for the treatment of chronic inflammatory rheumatism.

## Supplementary Information

**Additional file 1: Figure S1.** PPI network of GSE41038 DEGs.

**Additional file 2: Figure S2.** PPI network of GSE12021 DEGs.

**Additional file 3: Figure S3.** PPI network of GSE30023 DEGs.

**Additional file 4: Figure S4.** Venn diagram of the DEGs.

## Data Availability

The data used to support the findings of this study are included within the article.
