# Supplementary figures and images for: Comparison of immune cells and diagnostic markers between spondyloarthritis and rheumatoid arthritis by bioinformatics analysis
Source: J Transl Med. 2022 May 4;20:196. doi: 10.1186/s12967-022-03390-y (PMC9066892; doi:10.1186/s12967-022-03390-y)

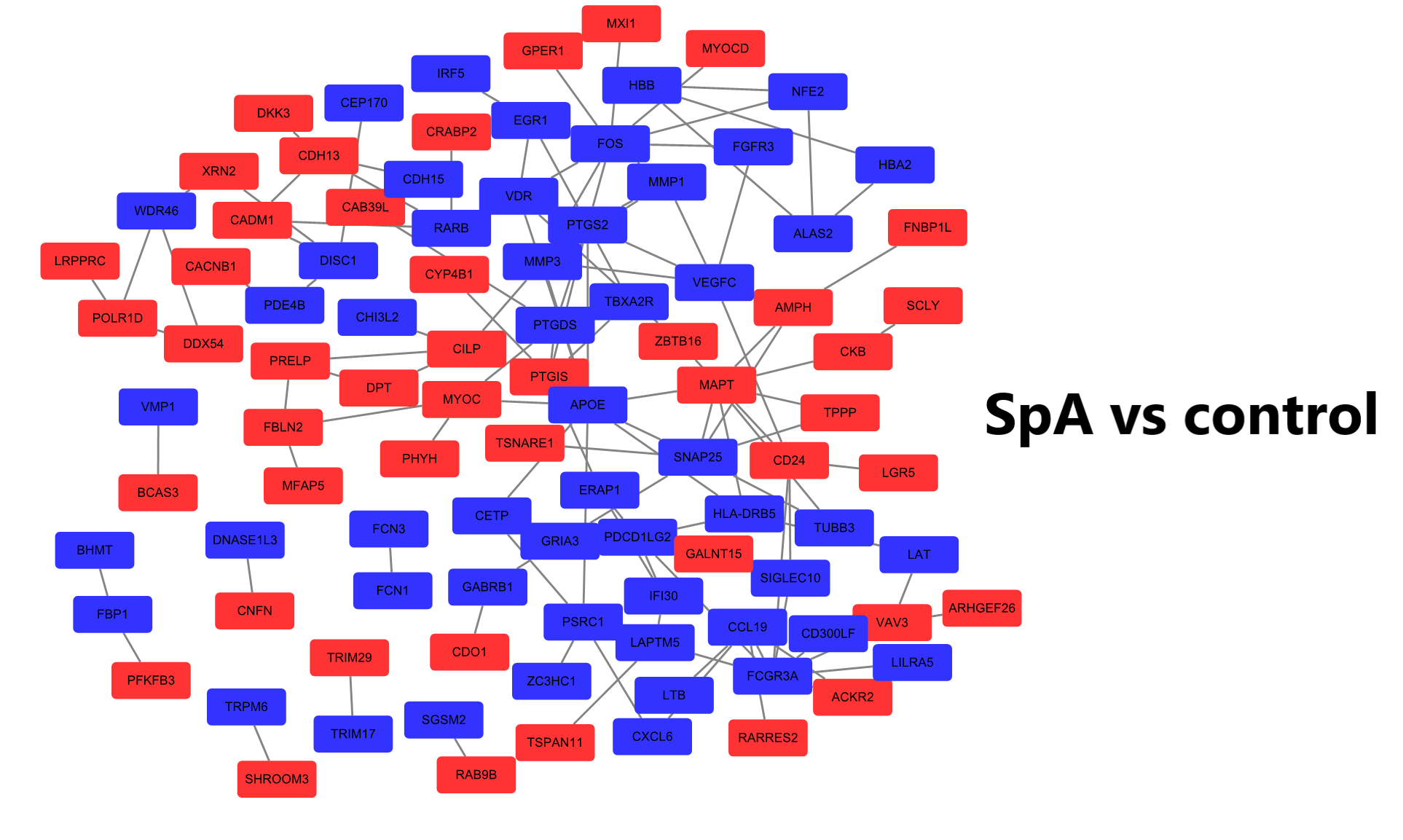

Supplement: Supplementary file 1 — Additional file 1: Figure S1. PPI network of GSE41038 DEGs. [file 12967_2022_3390_MOESM1_ESM.tiff]

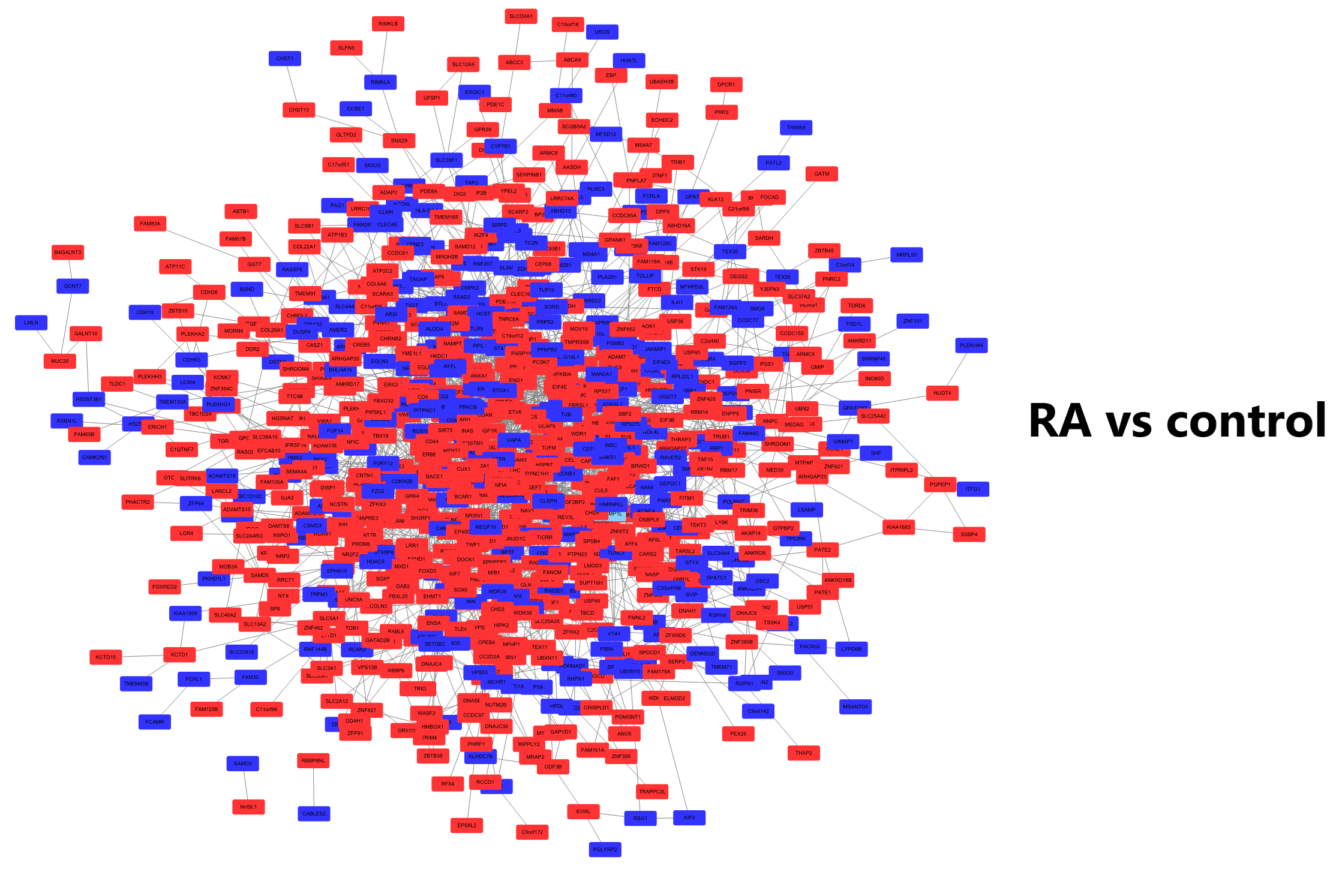

Supplement: Supplementary file 2 — Additional file 2: Figure S2. PPI network of GSE12021 DEGs. [file 12967_2022_3390_MOESM2_ESM.tiff]

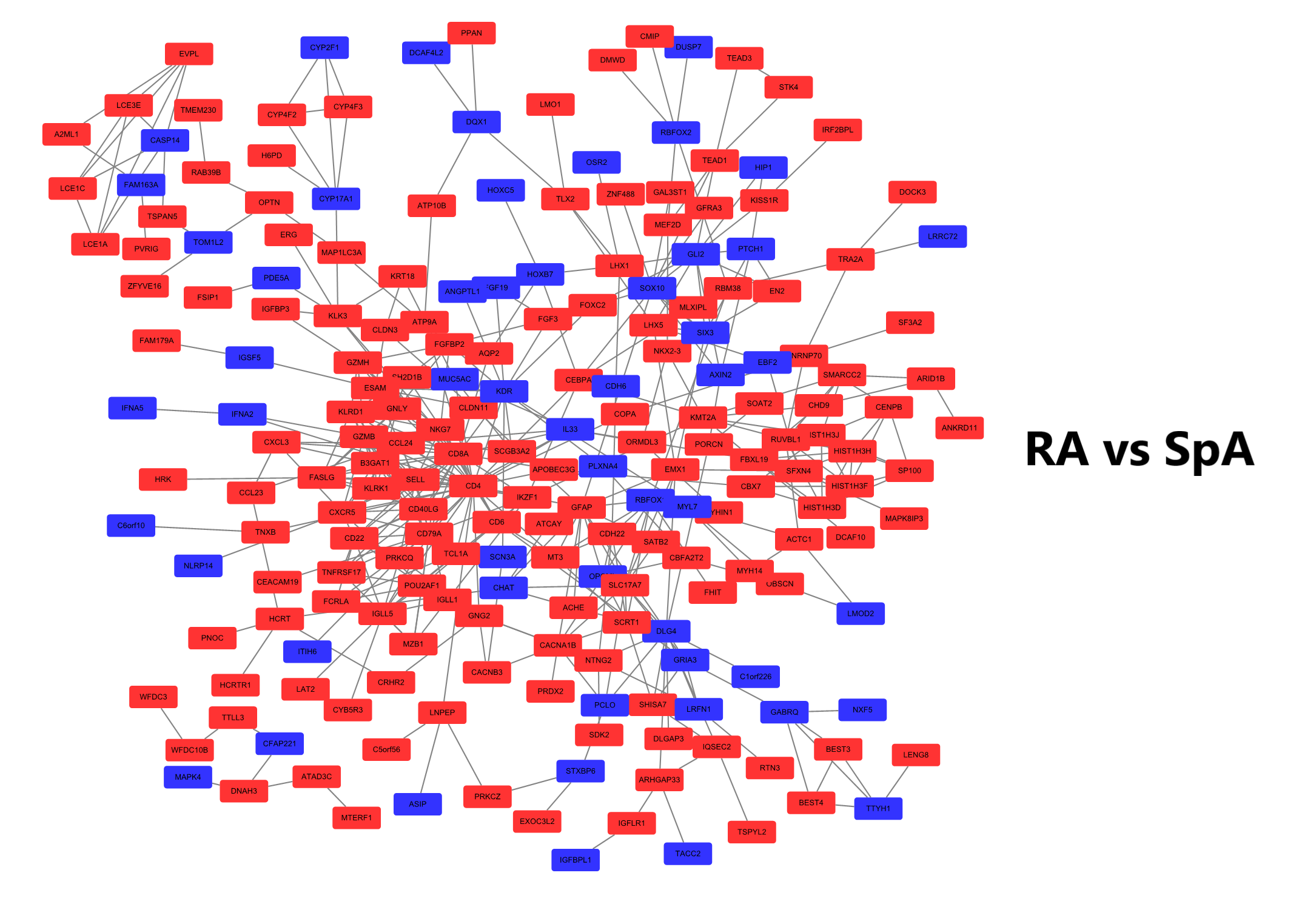

Supplement: Supplementary file 3 — Additional file 3: Figure S3. PPI network of GSE30023 DEGs. [file 12967_2022_3390_MOESM3_ESM.tiff]

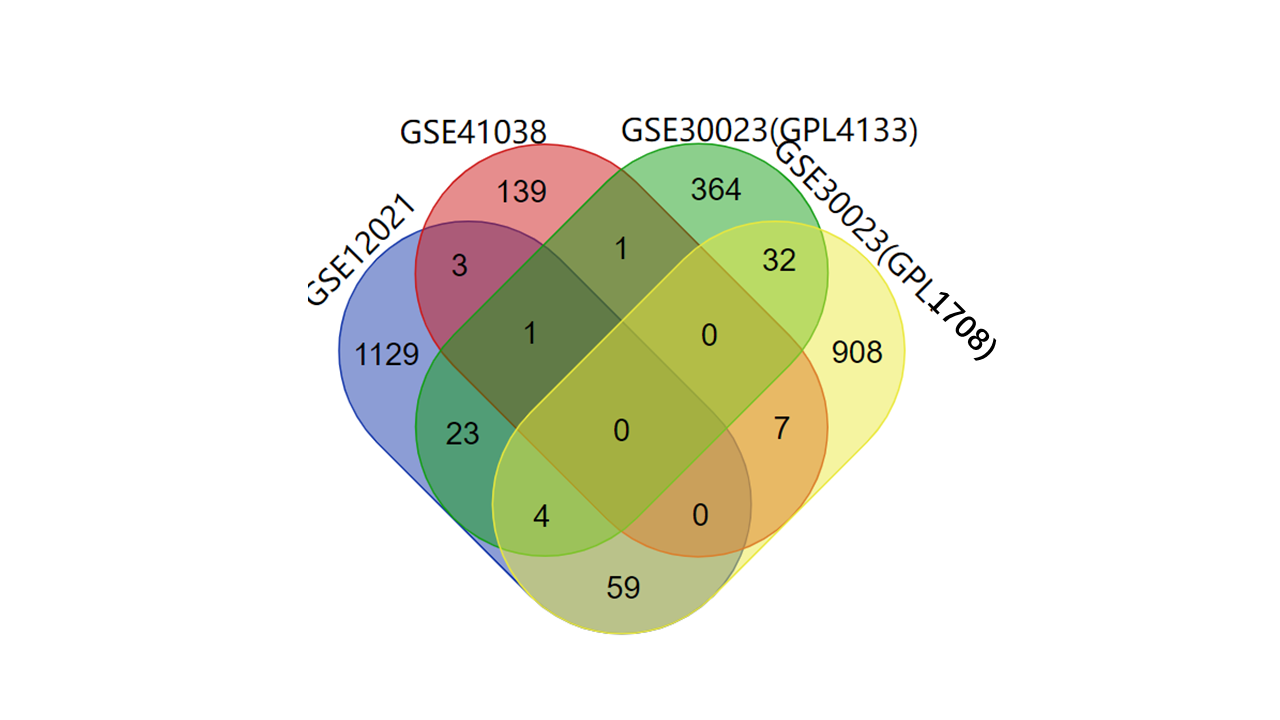

Supplement: Supplementary file 4 — Additional file 4: Figure S4. Venn diagram of the DEGs. [file 12967_2022_3390_MOESM4_ESM.tif]
